# Effect of Hydroxyapatite Nanoparticles on the Degradability of Random Poly(butylene terephthalate-*co*-aliphatic dicarboxylate)s Having a High Content of Terephthalic Units

**DOI:** 10.3390/polym8070253

**Published:** 2016-07-06

**Authors:** Nina Heidarzadeh, Mehdi Rafizadeh, Faramarz Afshar Taromi, Luís Javier del Valle, Lourdes Franco, Jordi Puiggalí

**Affiliations:** 1 Department of Polymer Engineering and Color Technology, Amirkabir University of Technology, Tehran 15875-441, Iran; nina_heidarzadeh@yahoo.com (N.H.); afshar@aut.ac.ir (F.A.T.); 2 Chemical Engineering Department, Polytechnic University of Catalonia, Av. Diagonal 647, Barcelona E-08028, Spain; luis.javier.del.valle@upc.edu (L.J.d.V.); lourdes.franco@upc.edu (L.F.)

**Keywords:** poly(butylene terephthalate), copolyesters, hydroxyapatite, nanocomposites, biocompatibility, hydrolytic degradation, enzymatic degradation

## Abstract

Copolyesters derived from 1,4-butanediol and constituted also of aliphatic and aromatic dicarboxylate units in a molar ratio of 3:7 were synthesized by a two-step polycondensation procedure. Succinic, adipic, and sebacic acids were specifically selected as the aliphatic component whereas terephthalic acid was chosen as the aromatic moiety. The second synthesis step was a thermal transesterification between the corresponding homopolymers, always attaining a random distribution as verified by NMR spectroscopy. Hybrid polymer composites containing 2.5 wt % of hydroxyapatite (HAp) were also prepared by in situ polymerization. Hydroxyl groups on the nanoparticle surface allowed the grafting of polymer chains in such a way that composites were mostly insoluble in the typical solvents of the parent copolyesters. HAp had some influence on crystallization from the melt, thermal stability, and mechanical properties. HAp also improved the biocompatibility of samples due to the presence of Ca^2+^ cations and the damping effect of phosphate groups. Interestingly, HAp resulted in a significant increase in the hydrophilicity of samples, which considerably affected both enzymatic and hydrolytic degradability. Slight differences were also found in the function of the dicarboxylic component, as the lowest degradation rates was found for the sample constituted of the most hydrophobic sebacic acid units.

## 1. Introduction

Polyesters are currently among the most competitive biodegradable polymers commercialized as both commodity and speciality products. Applications range from materials for solving “white pollution concerns” caused by non-degradable polymers to materials with good properties to fulfil, for example, the highly restrictive requirements of the biomedical field [1,2,3]. The most common polyesters are prepared by ring-opening polymerization of lactones (e.g., polyglycolide, polylactide, and poly(ɛ-caprolactone)) [4,5], but the production of poly(alkylene dicarboxylate)s has attracted a great deal of attention as well, especially for commodity applications [6].

Poly(butylene succinate) (PBS) is considered as one of the most important biodegradable poly(alkylene dicarboxylate)s commercialized up to now, because of its considerable combination of desired properties (e.g., high biodegradation rate, elastic modulus, and melting point) [7,8]. Such features generally worsen when another diol or aliphatic dicarboxylic acid is employed, making it very interesting to incorporate rigid units such as aromatic dicarboxylates to avoid any performance drop. Polyesters derived from aromatic dicarboxylic acids are much less electrophilic and, thus, far less sensitive to hydrolysis than aliphatic polyesters. However, aromatic polyesters may possess higher melting temperature (*T_m_*’s) and better mechanical properties. Therefore, it is an attractive challenge to incorporate aliphatic units randomly and determine if it is possible to optimize the properties in the direction of a satisfactory rate of biodegradation while retaining good mechanical properties. In the range of 30 mol % to 60 mol % of terephthalic acid, which is of particular interest since such materials provide useful material properties; the degradation rate drops linearly with the content of the aromatic acid [9,10]. Furthermore, these units may reduce the final cost of the polymer. Therefore, BASF and Novamont have commercialized aliphatic/aromatic polyesters derived from 1,4-butanediol and either adipic and terephthalic acids (i.e., Ecoflex™ and Origo-Bi™, respectively).

Biodegradation of these polymers by microorganisms or in a compost site is well proved, with the degradation process being promoted by a lipase-like hydrolase enzyme [11,12,13,14].

The biodegradation rate of aliphatic/aromatic polyesters depends on different interplaying factors such as: (a) chain microstructure (e.g., random or blocky disposition); (b) aromatic content; and (c) the degree of crystallinity [15,16,17,18,19,20,21]. For example, note that a comonomer content close to 50% and a random microstructure is expected to result in a decrease in the degree of crystallinity and logically an increase in the degradation rate.

The present work has been focused on aliphatic/aromatic polyesters derived from 1,4-butanediol, different aliphatic dicarboxylic acids (i.e., succinic, adipic, and sebacic acids) and a high content of terephthalic acid (i.e., a nominal value of 70 molar % with respect to the total dicarboxylic acid content was selected in order to explore the possibility of maintaining the biodegradable characteristics at such a high content). In this case, one should expect a low degradation rate as a result of such high aromatic content and the relatively high degree of crystallinity. Incorporation of hydrophilic hydroxyapatite (HAp) nanoparticles may increase both biodegradability and biocompatibility, and can be considered as an interesting option that will be evaluated for the proposed copolymers. To this end, copolymers with a 2.5 wt % of HAp will be synthesized as well.

HAp (Ca_10_(PO_4_)_6_(OH)_2_) is a bioceramic that forms part of the majority of the inorganic components of hard and connective tissues such as bones, teeth, and tendons. Nowadays, development of Hap-based nanocomposites and biodegradable polymers is attracting increasing interest since the presence of hydroxyl groups in the added nanoparticles may enhance polymer degradation. Furthermore, HAp particles can provide osteoconductivity and osteogravitivity as well as improved mechanical properties [22].

Hybrid HAp/biodegradable polymer composites can be prepared by different techniques, such as hot press moulding, plasma spraying, solution compounding, and also in situ polymerization, which is the most frequently employed method [23]. Distribution of nano-HAp particles in the organic matrix and final mechanical properties of the composite mainly depend on the interface compatibility between organic and inorganic phases. A good adhesion is expected when the hybrid is prepared by in situ polymerization, taking the capability of hydroxyl groups of the HAp surface to bind carboxyl groups of the growing poly(alkylene dicarboxylate) into account. Nevertheless, some limitations may also be expected as a consequence of the low concentration and reactivity of hydroxyl groups on the nanoparticle surface, which could lead to a reduced number of grafted molecules.

## 2. Experimental Section

Materials: Terephthalic acid (TA) was supplied by Shahid Toundgoyan Petrochemical Complex (Mahshar, Iran). Nanohydroxiapatite (n-HAp) was purchased from Beijing DK Nano Technology Co., Ltd., Beijing, China. 1,4-butanediol (BDO), sebacic acid (SeA), adipic acid (AdA) and succinic acid (SuA) were bought from Daejung Chemical & Metal Co., Ltd., Shiheung, Korea. Titanium butoxide (TBT) as polycondensation catalyst was purchased from Merck Co. (Darmstadt, Germany).

African green monkey kidney fibroblast-like (COS-7) and epithelial-like Madin–Darby Canine Kidney (MDCK) cells (ATCC, Manassas, VA, USA) were used in this work. Fetal bovine serum (FBS) was purchased from Gibco (Thermo Fisher Scientific Inc., Alcobendas, Spain). Lipases from pancreas porcine and *Pseudomonas cepacia* were purchased from Sigma Aldrich (Barcelona, Spain).

Polymerization: Synthesis of the three selected copolymers named as polybutylene succinate-*co*-terephthalate (PBST), poly(butylene adipate-*co*-terephthalate) (PBAdT) and poly(butylene sebacate-*co*-terephthalate) (PBSeT) was performed in a home-made 1-L high-pressure reactor with effective stirring, following a two-step melt polycondensation procedure.

(a) Synthesis of prepolymers from 1,4-butanediol and the selected dicarboxylic unit. Polycondensation reactions were carried out using an excess of 1,4-butanediol (BDO) (i.e., 1.7:1 [OH]:[COOH]). After loading the reactor with 650 g of a mixture of BDO and the appropriate dicarboxylic acid, the reaction mixture was firstly stirred for 30 min at 140 °C under a pressure of 3–3.5 bars. A flow of N_2_ was provided to keep the required pressure while an electric condenser allowed separating the condensed water and the excess of alcohol. Reaction temperature was then increased to 215, 207, 200, and 220 °C, for sebacic, adipic, succinic, and terephthalic acid derivatives, respectively. The reaction was stopped when no more water could be recovered (approximately after 180 min). In fact, the reaction extent could be evaluated by weighing the water that was recovered at regular time intervals.
(b) Vacuum polycondensation and thermal transesterification between aliphatic and aromatic prepolymers. The appropriate mixtures constituted by a 0.3:0.7 molar ratio of prepolymers derived from the aliphatic and aromatic acids and TBT as catalyst (1.4 mmol for 1 mol of dicarboxylic acid) were transferred to the reactor for 10 min at 200 °C and atmospheric pressure. Temperature was subsequently increased up to 250 °C while a vacuum (of 20 mbar) was applied. Once the mixer torque reached the desired value, the reaction was stopped (approximately after 150 min). Copolymers were dissolved in 1,1,1,3,3,3-hexafluroisopropanol (HFIP), precipitated in water, washed several times with water, methanol, and ether, and finally dried in a vacuum desiccator.

The chosen procedure differs from the conventional syntheses based on thermal polycondensation processes from an appropriate ratio of comonomers. The advantage of the proposed synthesis is that copolymers with different composition (i.e., having different comonomer ratios) can be easily obtained if desired from the corresponding homopolymers. Nevertheless, the time and reaction temperature of the second step appear highly important to achieve a random microstructure that should be checked.

Nanocomposites were synthesized based on the same protocol, except for the addition of a 2.5 wt % n-HAp at the beginning of copolymerization step. Nanocomposites will be referred to as PBST-HAp, PBAdT-Hap, and PBSeT-HAp depending on the aliphatic dicarboxylic unit.

Measurements: Molecular weights were estimated by size exclusion chromatography (GPC) using a liquid chromatograph (Shimadzu, model LC-8A, Tokyo, Japan) equipped with an Empower computer program (Waters, Milford, MA USA). A PL HFIP gel column (Polymer Lab, Böblingen, Germany) and a refractive index detector (Shimadzu RID-10A, Tokyo, Japan) were employed. The polymer was dissolved and eluted in HFIP containing CF_3_COONa (0.05 M) at a flow rate of 1 mL/min (injected volume 100 μL, sample concentration 2.0 mg/mL). The number and weight average molecular weights were calculated using polymethyl methacrylate standards.

Carboxylic acid end-group content was calculated, 1 g of polymer was dissolved in 20 mL chloroform and titrated using 0.05 N KOH in methanol with red phenol as the indicator. The carboxylic acid content [COOH] in meq/kg was measured using the following equation:

(1) $$[\text{COOH}]=\frac{(\upsilon -\upsilon_{0})\times m\times 1000}{w}$$

where *v* and *v*_0_ are the volume of the titrant (mL) for titration of the chloroform solution with and without the analyte, respectively, *m* is the titrant concentration (N), and *w* is the weight (g) of the polymer sample.

Infrared absorption spectra were recorded in the 4000–600 cm^−1^ range with a Fourier Transform FTIR 4100 Jasco spectrometer (Jasco International Co. Ltd., Tokyo, Japan) dotted with a Specac model MKII Golden Gate attenuated total reflection (ATR) cell.

^1^H NMR spectra were acquired with a Bruker AMX-300 spectrometer (Bruker Co., Bremen, Germany) operating at 300.1 MHz. Chemical shifts were calibrated using tetramethylsilane as an internal standard. A mixture (1:1 *v*/*v*) of deuterated chloroform and trifluoroacetic acid (TFA) was used as the solvent.

Calorimetric data were obtained by differential scanning calorimetry with a TA Instruments Q100 series (TA Instruments, New Castle, DE, USA) with *T*_zero_ technology equipped with a refrigerated cooling system (RCS). Experiments were conducted under a flow of dry nitrogen with a sample weight of approximately 5 mg and calibration was performed with indium. *T*_zero_ calibration required two experiments: the first experiment was performed without any sample while in the second case sapphire disks were used. A first heating run (20 °C/min) was performed to determine melting temperature and enthalpy of the as-synthesized sample. To erase the thermal history the sample was then kept in the melt state for three minutes and subsequently cooled to room temperature (10 °C/min) to obtain crystallization data. Finally, a second heating run (20 °C/min) was carried out to characterize the melt crystallized sample.

Thermal degradation was studied at a heating rate of 10 °C/min with around 5 mg samples in a Q50 thermogravimetric analyser (TGA) of TA Instruments, under a flow of dry nitrogen. Test temperatures ranged from 50 to 600 °C.

A TA Instruments DMA Q800 (TA Instruments, New Castle, DE, USA) was used to study the dynamic mechanical properties of materials. Prismatic rectangular samples (ca. 10 × 12 × 1.3 mm^3^) were analysed in single-cantilever mode at 1 Hz and 10 μm strain amplitude at 3 °C/min from −100 to 100 °C.

Contact angles (CA) were measured at room temperature with sessile drops using an OCA-15 plus Contact Angle Microscope (DataPhysics Instruments GmbH, Filderstadcity, Germany) and SCA20 software. Contact angle values of the right and left sides of distilled water drops were measured and averaged. Measurements were performed 10 s after the drop (0.5 µL) was deposited on the sample surface. All CA data were obtained by averaging between six measurements on different surface locations.

Distribution of HAp nanoparticles in the composites was evaluated by means of a Philips TECNAI 10 electron microscope (Philips Electron Optics, Eindhoven, The Netherlands) at an accelerating voltage of 80 kV. Samples were prepared by embedding the nanocomposite specimens. A low viscosity modified Spurr epoxy resin was employed to embed the specimens before curing and cutting in small sections. In this case, a Sorvall Porter-Blum microtome (Norwalk, CT, USA) equipped with a diamond knife was utilized. The thin sections were collected in a trough filled with water and lifted onto carbon coated copper grids.

X-ray diffraction patterns were acquired using a Bruker D8 Advance model (Bruker, Karlsruhe, Germany) with Cu K_α_ radiation (λ = 0.1542 nm) and the geometry of Bragg–Brentano, theta–2theta. A one-dimensional Lynx Eye detector (Bruker, Karlsruhe, Germany) was employed.

Degradation studies: Thermally moulded films were used for biocompatibility and degradation studies. Briefly, polymers were heated up to 25 °C above their melting point for 2 min using a hydraulic press equipped with heating plates and a temperature controller (Specac, Orpington, UK). Pressure was progressively increased to 3–4 bars. Polymer films with a thickness of about 150 µm were recovered after cooling the mould to room temperature and subsequently cut to the desired size.

Cell adhesion and proliferation assays: COS-7 and MDCK cells were cultured in Dulbecco’s modified Eagle medium (DMEM) as previously reported [24]. Square pieces (10 × 10 × 0.15 mm^3^) of press molten films were placed and fixed in each well of a multi-well culture plate with a small drop of silicone (Silbione^®^ MED ADH 4300 RTV, Bluestar Silicones France SAS, Lyon, France). They were then sterilized by UV radiation in a laminar flux cabinet for 15 min. Thereafter, the samples were stabilized for 24 h in 1 mL of medium under culture conditions. For cell adhesion and proliferation assays, aliquots of 50–100 μL containing 5 × 10^4^ and 2 × 10^4^ cells, respectively, were seeded onto the films in each well containing 1 mL of medium, and incubated for 24 h (adhesion assay) or seven days (proliferation assay). Samples were evaluated by the standard adhesion and proliferation method [24]. The used procedure is based on a simple modification of the ISO10993-5:2009 standard test that describes the appropriate methodology to assess the in vitro cytotoxicity of medical devices. Finally, the cellular viability on materials was evaluated through the MTT assay [24]. The study was carried out using five replicates and the results were averaged. Samples with adhered and grown cells on the films were fixed with 2.5% *w*/*v* formaldehyde at 4 °C overnight. They were subsequently dehydrated and processed for observation via scanning electronic microscopy.

In vitro hydrolytic degradation assays were carried out in a pH 7.4 phosphate buffer (19.268 g of Na_2_HPO_4_·12H_2_O and 1.796 g of KH_2_PO_4_ in 1 L of deionized water) at 37 °C and at the accelerated condition provided by raising temperature to 70 °C. Samples (2 × 2 cm^2^ square pieces) were kept under orbital shaking in bottles filled with 20 mL of the degradation medium and sodium azide (0.03 wt %) to prevent microbial growth for the selected exposure times. The samples were then thoroughly rinsed with distilled water, dried to constant weight under a vacuum, and stored over P_4_O_10_ before analysis. Degradation studies were performed in triplicate and the results were averaged.

Enzymatic studies were carried out with lipases from a porcine pancreas *(*56–60 U/mg) and *Pseudomonas cepacia* (≥30 U/mg) using three replicates. All samples (1 × 1 cm^2^ square pieces) were exposed to 2 mL of pH 7.4 phosphate buffer at 37 °C. The buffer contained the enzyme along with sodium azide (0.03% *w*/*v*) and calcium chloride (5 mM). Solutions were replaced every 48 h to prevent enzymatic activity loss. Samples were extracted, washed, and dried as indicated before.

Scanning electron microscopy (SEM) was utilized to examine the morphology of films after different times of exposure to the selected degradation media. Carbon coating was accomplished with a Mitec k950 Sputter Coater (fitted with a film thickness monitor k150x) (Quorum Technologies Ltd., West Sussex, UK). SEM micrographs were obtained with a Zeiss Neon 40 EsB instrument (Carl Zeiss, Oberkochen, Germany).

Weight retention (*W_r_*) of the specimens was addressed by the percentage ratio of weight after degradation (*W*_d_) to initial weight before degradation (*W*_0_):

(2) $$W_{r\;}=\;W_{d\;}/\;W_{0\;}\times 100$$

## 3. Results and Discussion

### 3.1. Synthesis and Characterization of Copolymers and Nanocomposites

All copolymers were obtained with a high yield that ranged between 85% and 90%; no specific trend concerning the length of the aliphatic copolymer or the incorporation of HAp nanoparticles was observed. The carboxyl content was always below 50 meq, indicating that degradation during polymerization step was not significant despite the fact that the recovered solid had a slightly brown/grey appearance. Nevertheless, white products were obtained after purification. Weight average molecular weights of purified copolymers varied between 17,100 g/mol and 20,200 g/mol, and the polydispersity index remained in the typical range of polycondensation samples (i.e., between 2.1 and 2.4) (Table 1).

**Figure 1:**
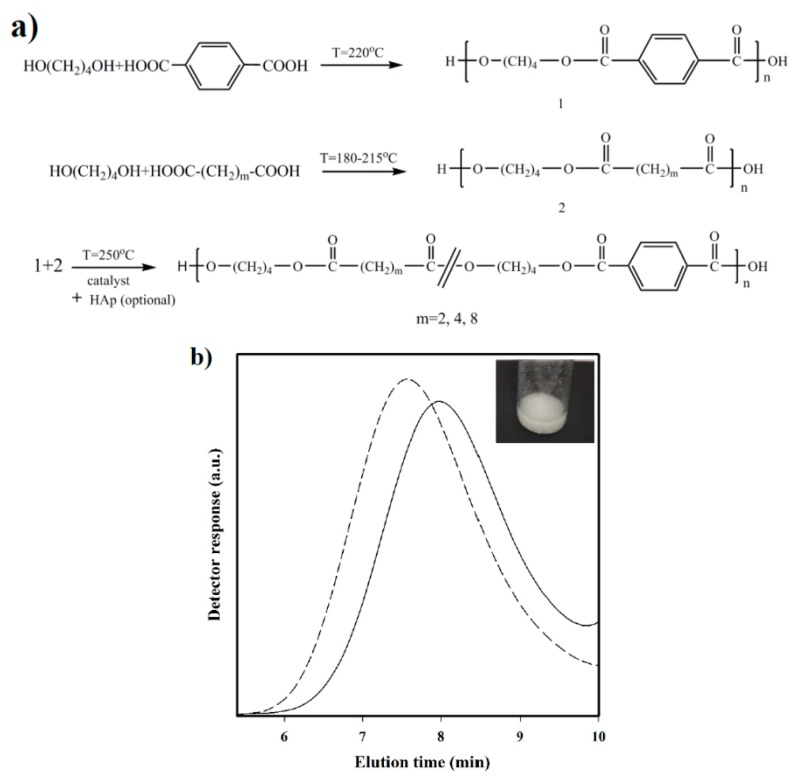
(**a**) Synthesis scheme for different studied copolyesters and nanocomposites; (**b**) GPC curves of PBST (solid line) and PBST-HAp (dashed line) samples. The inset shows a photograph of a highly concentrated suspension of PBST-HAp in HFIP.

Nanocomposites prepared by in situ polymerization were highly insoluble and a high proportion remained as a whitish particle dispersion in the HFIP solvent used for GPC measurements (inset of Figure 1). This feature indicates a significant grafting as the consequence of reaction between hydroxyl groups on the HAp surface with terminal carboxylic groups of the growing copolymers. Nevertheless, a small fraction corresponding to the lower molecular weight chains (i.e., those that were practically ungrafted) could be dissolved in HFIP at a low concentration. In this case, *M*_w_ values ranged between 22,200 g/mol and 26,170 g/mol, with the molecular size always greater than the size determined for the corresponding copolymers. The greatest difference was found in the case of the adipic derivative (i.e., from 17,070 g/mol to 26,170 g/mol) and in particular, GPC curves showed a peak broadening and shift for the nanocomposite (Figure 1) due to the presence of some grafted chains. In fact, the polydispersity index of the three nanocomposites was always greater (i.e., 2.4–2.7 with respect to 2.1–2.4) than that determined for copolymers, indicating a wider size distribution that reflects the presence of a small ratio of cross-linked chains. Note that a lower molecular weight and a narrow distribution should be expected if only the smallest and non-grafted chains were dissolved.

**Figure 2:**
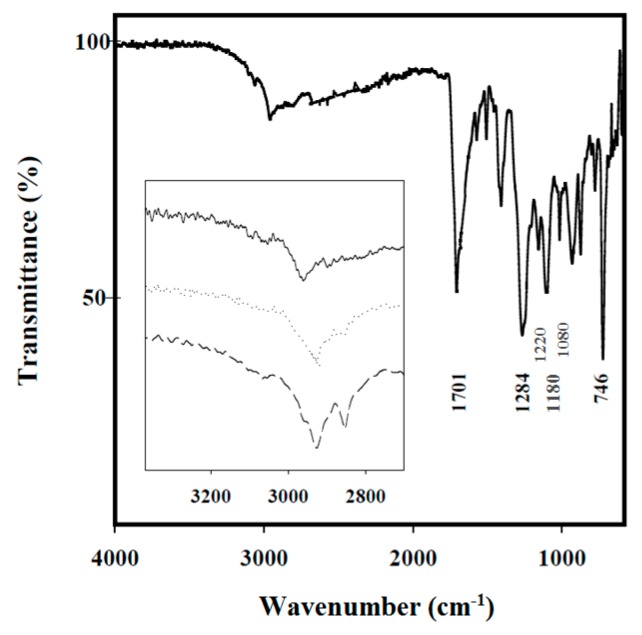
FTIR spectrum of PBST-HAp. The inset compares the methylene bands of the three copolymers when spectra were normalized with the intensity of the C=O band.

FTIR spectra of copolymers and nanocomposites showed the characteristic absorption bands for methylene (2930 and 2850 cm^−1^, stretching), C=O (1701 cm^−1^, stretching), aromatic C–O (1284 and 1180 cm^−1^, asymmetric and symmetric stretching, respectively), aliphatic C–O (1220 and 1080 cm^−1^, asymmetric and symmetric stretching, respectively), and aromatic (746 cm^−1^) groups. A representative spectrum of PBTSu-HAp is illustrated in Figure 2. As the length of the aliphatic comonomer increased, the intensities associated with methylene groups were logically increased compared to those related to the C=O group (inset of Figure 2). The bands corresponding to HAp could not be detected in the spectra of different composites due to the low loaded percentage of HAp.

**Figure 3:**
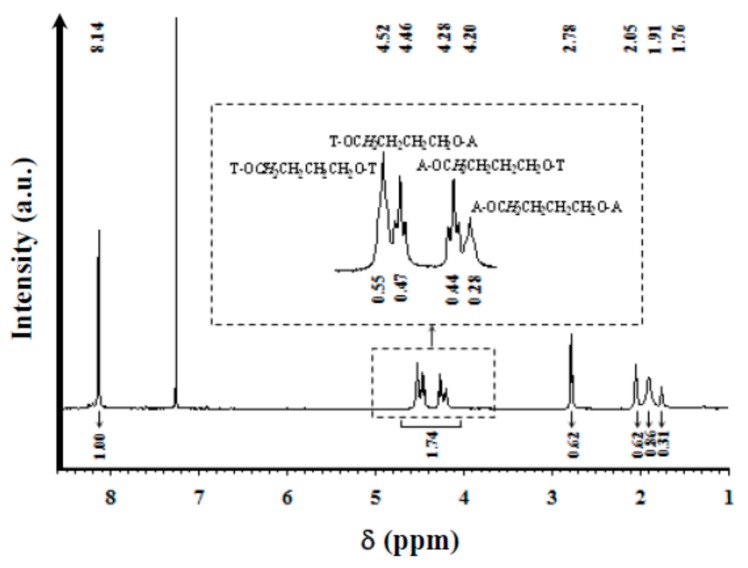
^1^H NMR spectrum of PBST. The inset shows a magnification of the 4.70–4.25 ppm region where OC*H*_2_ sequence sensitive signals appear.

^1^H NMR spectra were useful for determining the composition and performing an analysis of sequences, as previously reported for adipic acid copolymers [18]. Figure 3 shows the spectrum for the representative PBST copolymer. Table 2 summarizes the peak assignment for the three copolymers, whereas the analysed data are given in Table 3. The areas of signals at 8.14–8.17 ppm (terephthalate units, *T*) and 2.42–2.79 ppm (aliphatic dicarboxylate units, A) were used to determine the corresponding (*f*_T_ and *f*_A_) mole fractions.

The OC*H*_2_ butanediol protons were highly sensitive to the neighbouring dicarboxylic acid units and consequently four multiplets were detected for all studied samples (Table 2).

The respective areas were used to determine the fractions corresponding to TBT (*f*_TT_), TBA (*f*_TA_), ABT (*f*_AT_), and ABA (*f*_AA_) sequences. Obviously *f*_TA_ and *f*_AT_ should be equal, being the summarized values averaged from the intensities of TCH_2_..A and ACH_2_…T signals. These values allowed for determining the probability of finding a T unit next to an AB sequence (*P*_AT_), as well as the probability of finding an aliphatic unit next to a TB sequence (*P*_TA_):

*P*_AT_ = *f*_AT_/*f*_A_ (3)

*P*_TA_ = *f*_TA_/*f*_A_ (4)

Finally, the block lengths of the AB and TB sequences were calculated as:

*L*_nAB_ = 1/*P_AT_* (5)

*L*_nTB_ = 1/*P_TA_* (6)

The degree of randomness (*r*) is defined as the summation of the two probabilities (P_AT_ and *P*_TA_), in which the values 2, 1, and lower than 1 are indicative of alternating, random, and blocky distributions, respectively. The limit value of 0 logically indicates a mixture of the two homopolymers. Values summarized in Table 3 indicate a composition close to the theoretical one, although copolymers were slightly impoverished on the aromatic units probably as a consequence of the lower molecular weight and the higher content in butanediol units of terephthalate prepolymers, which caused a distortion in the calculation of theoretical feed ratio for the second synthesis step. Results also demonstrated that the three samples were associated with degree of randomness values close to 1.0, indicating a perfect statistical distribution. In fact, *f_TA_* and *f_AT_* values were consistent with those expected for a random distribution (e.g., 0.21 with respect to 0.22–0.25). Namely, transesterification reactions between prepolymers of each dicarboxylic acid should take place at a high reaction temperature, thereby hindering the possibility of achieving a blocky structure.

### 3.2. Thermal Properties of Copolyesters and Nanocomposites

**Figure 4:**
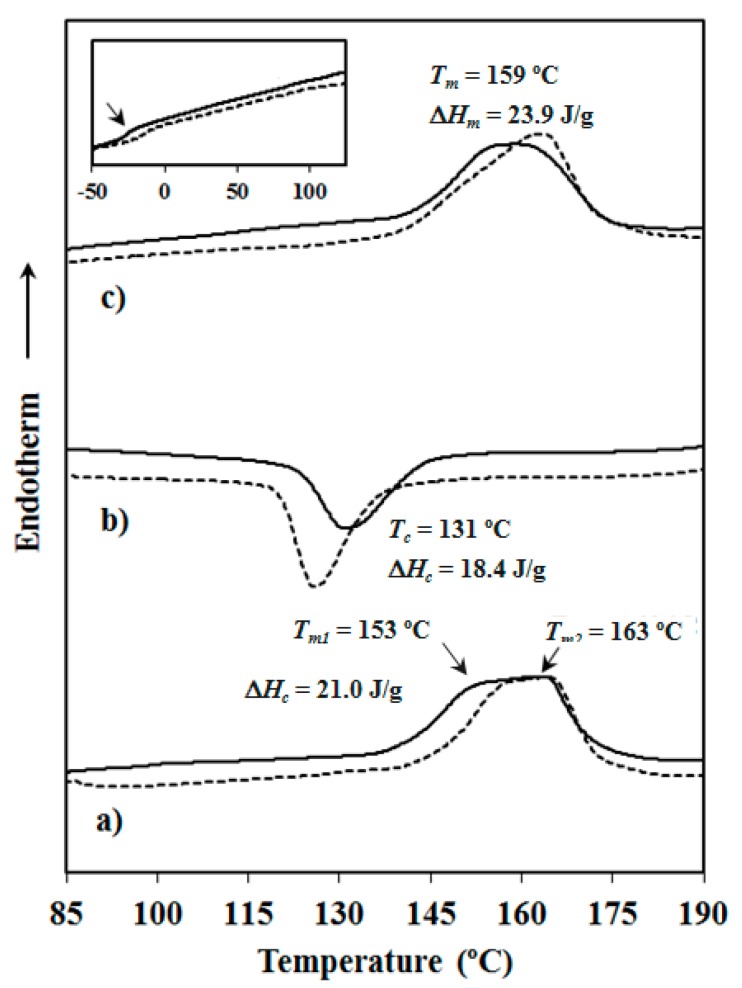
Typical sequence of DSC scans performed with PBAdT (solid lines) and PBAdT-HAp (dashed lines): (**a**) The initial heating scan of the as-synthesized sample; (**b**) the cooling run from the melt state; and (**c**) the heating run of the melt crystallized sample. Calorimetric data are only given for the sample without HAp. Inset shows the low temperature region for (**c**) in which *T_g_*s could be detected (arrow).

Figure 4 shows the typical protocol followed to determine the thermal properties of the synthesized samples, taking PBAdT and PBAdTA-HAp as representative examples, whereas DSC data for all studied specimens are summarized in Table 4.

In all cases, the first heating run showed a complex melting behaviour where two endothermic peaks appeared overlapped in practice. These melting temperatures were similar for all samples since fusion always corresponded to crystals predominantly constituted by butylene terephthalate sequences. Nevertheless, melting temperatures were clearly lower (i.e., by approximately 60 °C) than that reported for the PBT homopolymer (223 °C) [25]. Thus, a similar amount of aliphatic comonomer units seems to be incorporated into the crystals as could be expected by considering their similar composition (i.e., 30 molar % of aliphatic dicarboxylic units). DSC scans suggest the occurrence during heating of a typical recrystallization process where thickening of lamellae took place, giving rise to a high-temperature melting peak. Stability and thicknesses of lamellar crystals depend on the crystallization process. Thus, slight differences (i.e., peak temperatures and intensities) can be found between the first (i.e., solution crystallized sample) and the second heating (i.e., melt crystallized sample) runs, as shown in Figure 4 for the PBAdT copolyester. Melting enthalpies of all samples were also similar, and no significant difference was observed when the aliphatic dicarboxylic acid was changed or HAp was incorporated. Random copolyesters had a moderate crystallinity as estimated from the composition and the melting enthalpy of 100% crystalline PBT (142 J/g) [26].

The effect of HAp was only remarkable in the case of the adipic acid derivative since in this case the glass transition temperature was significantly increased (i.e., from −27 °C to −14 °C) and the crystallization process required a higher supercooling (Figure 4b), probably as a consequence of its higher molecular weight. Crystallization of the nanocomposite rendered more defective or thinner lamellae, as could be deduced by the decrease in both temperature and intensity of the first melting peak (Figure 4c). Namely, a recrystallization process during the second heating run was enhanced, giving rise to a greater ratio of thicker lamellae and a higher intensity of the corresponding melting peak.

**Figure 5:**
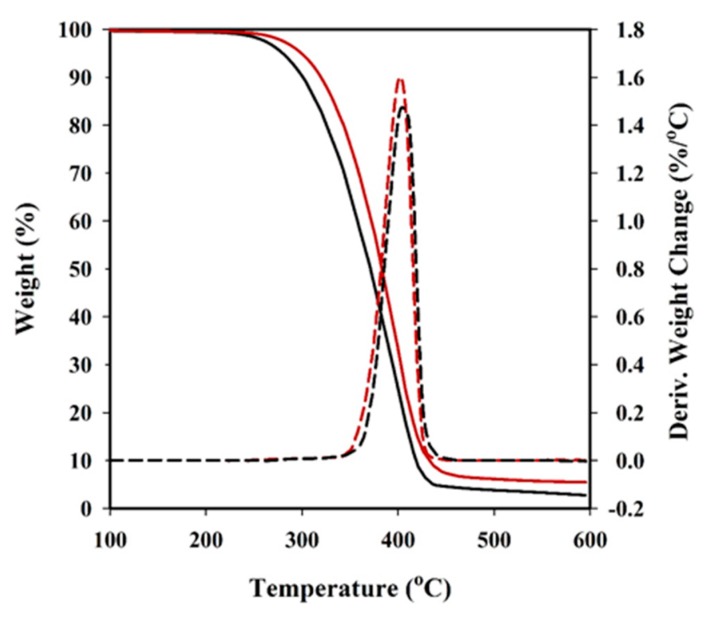
TGA (solid lines) and DTGA (dashed lines) curves comparing the thermal degradation of PBSuT (black lines) and PBSuT-HAp (red lines) samples.

TGA and DTGA traces showed that all studied samples thermally degraded at a temperature clearly higher than the corresponding melting points. Although decomposition seems to proceed as a single step (e.g., Figure 5 for PBST), different mechanisms are probably involved, as reported for PBT [27]. In this case, degradation started with an ionic decomposition process that results in the production of tetrahydrofuran. The subsequent process was associated with ester pyrolysis reactions that yield 1,3-butadiene at the beginning and finally produced aromatic species [27]. Thermal degradation of several poly(butylene succinate-*co*-butylene terephthalate)s has also been previously reported [28], indicating a single weight-loss step. Nevertheless, it was found that the aliphatic poly(butylene succinate) exhibited lower degradation temperature and thermal stability compared to PBT. The differences between our results and the previously reported data on the copolymer could be attributed to distinct chain microstructures due to alterations in the copolymerization process. Therefore in this study, the high-temperature step was performed with a prepolymer mixture instead of a monomer mixture. Data summarized in Table 5 indicate similar degradation behaviour for succinic, adipic, and sebacic acid derivatives.

Curves for nanocomposites progressed in a similar way but the incorporation of HAp had two main effects: (a) The DTGA peak associated with the first decomposition step was shifted by approximately 3 °C to higher temperatures. The observed stabilization may be a consequence of the reduction of free carboxylic groups due to the grafting reaction with the hydroxyl groups of HAp surface or even a hindered diffusion of degradation products. (b) The char yield logically increased to a value that was fully consistent with the theoretical amount of incorporated HAp (e.g., from 2.8% to 5.6% for the succinic derivative).

### 3.3. X-ray Diffraction Data

**Figure 6:**
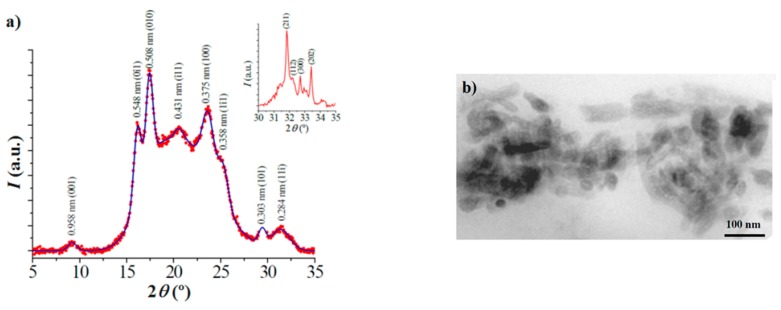
(**a**) X-ray diffraction profile of PBSTS-HAp with indication of the spacing of main reflections. The inset shows a magnification of the 2θ region between 30° and 35° where the strongest reflections of HAp appear; (**b**) Transmission electron micrograph of a thin section of PBTSu-HAp.

Powder X-ray diffraction patterns of all synthesized samples were similar and logically related to the structure of poly(butylene terephthalate). In particular, strong (001), (0-11), (010), (1-11), (100), (1-11), (101), and (11-1) reflections of the α-form defined by a triclinic unit cell (*a* = 0.483 nm, *b* = 0.596 nm, *c* = 1.162 nm, α = 99.9°, β = 115.2°, and γ = 113.8°) and a gauche-trans-gauche conformation for the glycol unit were observed (Figure 6) [29,30]. It is clear that copolymers crystallized according to the structure of the major component and that aliphatic comonomers should be mainly excluded from the crystalline phase, being probably located in the amorphous lamellar folds. Logically, the slight increase on the length of the aliphatic dicarboxylic unit should have a minor impact on the molecular arrangement of the crystalline phase. Patterns of nanocomposites also showed very low signals corresponding to HAp (inset of Figure 6a) related to the (211), (112), (300), and (202) reflections.

Transmission electron micrographs of nanocomposite thin sections revealed a relatively good dispersion of HAp thin crystals (Figure 6b). They showed a typical elongated hexagonal morphology with dimensions (length × width) ranging from 100 μm × 50 μm to 50 μm × 20 μm.

### 3.4. Mechanical Properties

**Figure 7:**
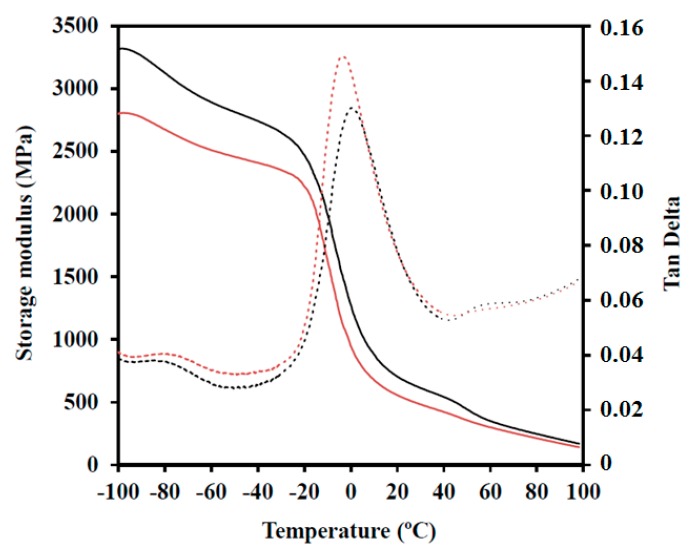
Storage modulus (*E*’) (solid line) and loss tangent (dashed line) curves of PBAdT (red) and PBAdT-HAp (black) samples.

The effect of HAp particles on mechanical properties was evaluated by DMTA, and the main results are summarized in Table 6. Storage modulus (*E*’) and loss tangent curves for representative PBAdT and PBAdT-HAp samples are shown in Figure 7. Some specific features that can be pointed out are as follows: (a) Storage moduli of nanocomposites are always greater than those measured for parent copolyesters (e.g., 2470 N/m^2^ with respect to 1840 N/m^2^ at −20 °C and 700 N/m^2^ with respect to 550 N/m^2^ at 20 °C for the adipic acid derivative); (b) the intensity of the loss tangent peak decreases for the nanocomposite indicating a more restricted chain mobility, which cannot be justified by the slight increase in crystallinity; (c) loss tangent curves show a predominant peak at a temperature close to 0 °C. This glass transition temperature only increased for the nanocomposite of the adipic acid and sebacic acid derivatives, with a very small observed variation (i.e., less than 5 °C).

### 3.5. Contact Angle Measurements

**Figure 8:**
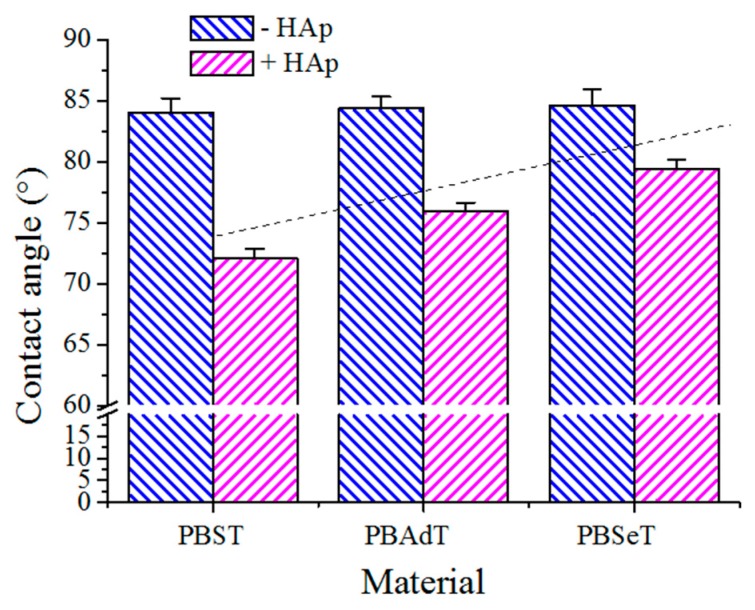
Contact angle measurements for water on films of PBST-HAp, PBAdT-HAp, PBSeT-HAp nanocomposites, and the corresponding HAp-free samples.

The contact angles of water droplets on films of the three copolymers were similar (i.e., between 83° and 85° as shown in Figure 8). Therefore, the change of the aliphatic dicarboxylic unit (i.e., the number of methylene groups) did not greatly affect the final hydrophobicity. In general, these surfaces could be considered in the borderline (90°) between hydrophobic and hydrophilic characters. It should be pointed out that the ratio of these aliphatic units was low (30% of the total dicarboxylic content) and probably a minimum influence of composition should be expected. On the contrary, a significant decrease up to 72–78° (Figure 8) was observed when HAp was incorporated. In this case, some differences were found for the three derivatives, as the hydrophobicity slightly increased with increase in the methylene content. The result is meaningful since degradability can be enhanced as a consequence of a higher hydrophilicity.

### 3.6. Cell Adhesion and Proliferation

**Figure 9:**
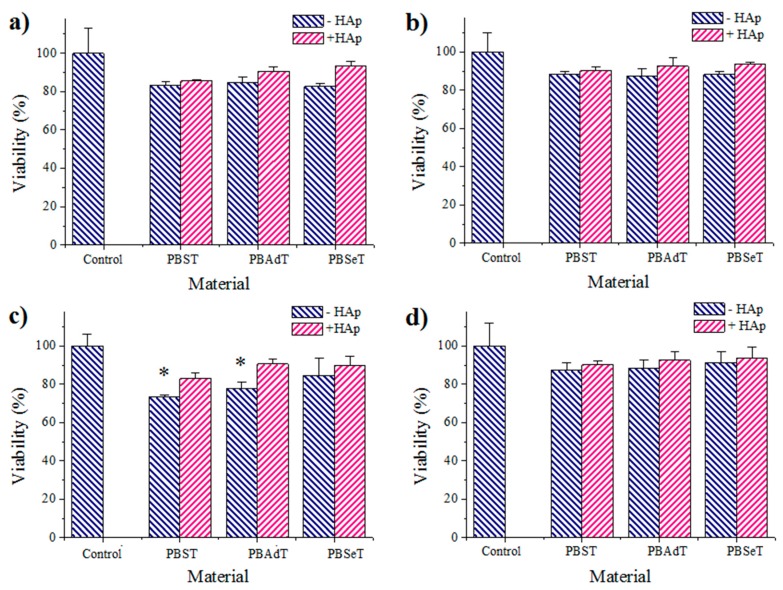
Adhesion (**a**,**c**) and proliferation (**b**,**d**) onto materials of: (**a**,**b**) COS-7 (fibroblast-like cells); (**c**,**d**) MDCK (epithelial-like cells). * *p* < 0.05 using Tukey test.

Figure 9 shows the adhesion and proliferation results corresponding to different samples, making use of both epithelial-like and fibroblast-like cell lines (MDCK and COS-7 cells, respectively). A similar cell adhesion of fibroblasts (close to 80%) was found for the three studied copolyesters, but, interestingly, it was slightly increased up to 90–95% when HAp was incorporated. The results are significant since they demonstrate that the biocompatible characteristics of PBT can be improved by the addition of HAp and that aliphatic dicarboxylic units may play an important role in such an observation. Note that the highest viability was detected for derivatives with longer adipic (90%) and sebacic acid (95%) units (Figure 9a). These results may be a consequence of the increased hydrophobicity of samples with longer aliphatic units.

Effects on adhesion were more evident when epithelial-like cells were employed since succinate and adipate compounds were associated with a statistically lower value (<80%) compared to the control, while the adhesion increased in the range of 80–95% for the sebacic acid derivative and also for all HAp composites (Figure 9c).

Experimental results could be explained by the fact that epithelial cells form a monolayer of flat cells with a polygonal/irregular shape and hence are in close contact, giving rise to apical and basolateral domains. In fact, the monolayer can be considered as a barrier between the material surface and the culture medium. Epithelial cells need a suitable material surface to adhere through the basal domain and consequently are highly sensitive to inappropriate surfaces. On the contrary, fibroblast cells are characterized with their high mobility (i.e., they can easily contract and extend) since do not form such a monolayer. Furthermore, there is no limitation on the interchange between the material surface and the medium due to the absence of apical and basolateral domains. Probably, polymers having short dicarboxylic units may cause an acidification of the culture medium and a reduction of viability. The incorporation of HAp may dampen this acidification since calcium phosphate can be considered as a buffering agent. On the other hand, it is well known that cellular adhesion is improved when Ca^2+^ and Mg^2+^ cations are present, the former being provided by HAp particles.

Proliferation results were similar for both fibroblast and epithelial cell lines (Figure 9b,d, respectively), a feature probably indicating that cell morphology is not crucial for long time (seven days) events. In general, results were satisfactory for all samples (viability in the 85–95% range), although a slight improvement was observed again when HAp was incorporated.

**Figure 10:**
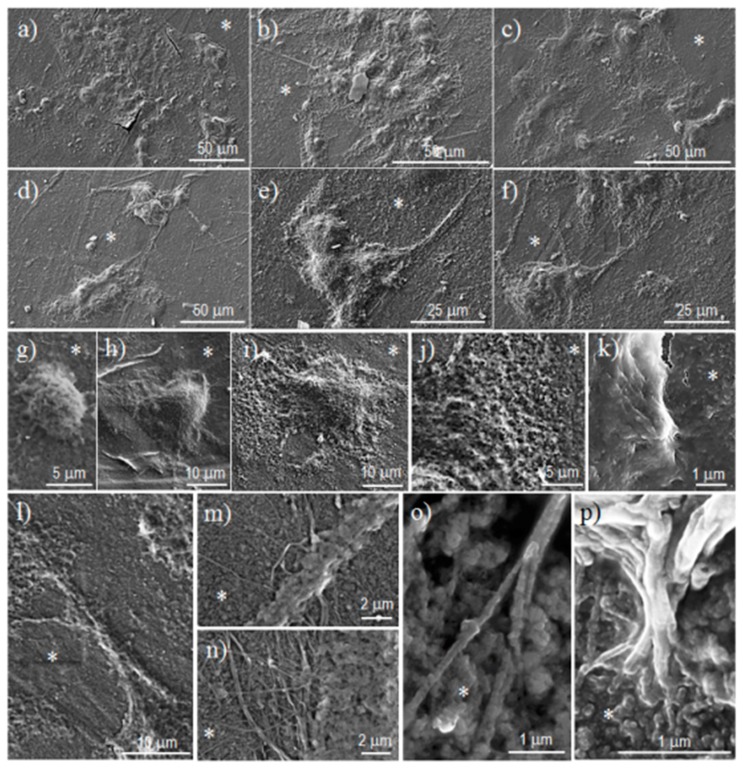
Epithelial-like MDCK cells adhered on PBST-HAp (**a**); PBAdT-HAp (**b**); and PBSeT-HAp (**c**); Fibroblast-like COS-7 cells adhered on PBST-HAp (**d**); PBAdT-HAp (**e**); and PBSeT-HAp (**f**). Overall details of the cellular adhesion of epithelial-like cells (**g**–**k**) and fibroblast-like cells (**l**–**p**). * indicates the material surface.

These results are qualitatively shown in Figure 10. The epithelial cells form monolayers that were organized into cellular clusters on the surface of the three copolymers (Figure 10a–c). Fibroblasts are more segregated on the surface of the copolymers and their cytoplasm extends with fusiform shapes (see arrows in Figure 10d–f). These characteristics of cell–surface interactions are typical for each cell type when the cytoskeleton is reorganized. Figure 10g–k show epithelial cells from an early interaction (as evidenced by its spherical shape) with the copolymer surface so as to form lamellipodia by extending their cytoplasm. Cells tended toward a flat shape and their base was fully extended on the copolymer surface (Figure 10i). This widespread adherence to the surface was achieved by multiple tiny hairs (<1 µm) that emerged from the membrane (Figure 10k). In contrast, fibroblast cells attach to the copolymer surface in a more localized manner. Long cytoplasmic extensions allowed for colonizing a large area of the material surface while interacting with other cells (Figure 10l). However, it is clear that multiple filopodias of different lengths (clearly >1 µm) emerged from the cytoplasmic extensions (Figure 10m,n). These filopodia were multi-filaments that become separated when interacting with the material. The finger-like structures (Figure 10o,p) gave rise to a good adhesion by appropriate anchoring onto the material.

### 3.7. Hydrolytic Degradation

**Figure 11:**
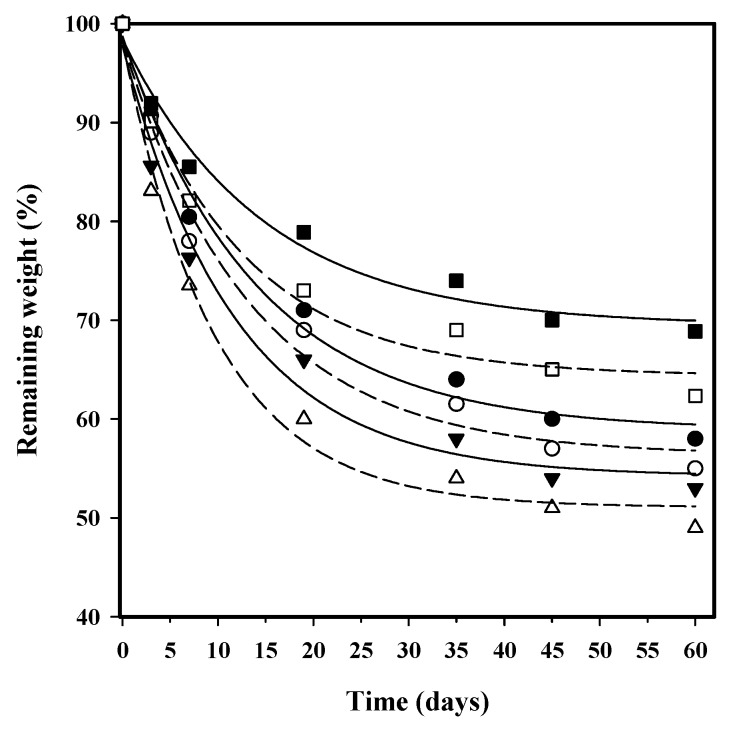
Plot of the variation of the remaining weight percentage during exposure time to a pH 7.4 hydrolytic medium at 70 °C for succinic (circles), adipic (triangles), and sebacic (squares) acid copolyesters with (open symbols) and without (full symbols) HAp.

Figure 11 shows the evolution of the remaining weight percentage of the studied copolyester films during exposure to a pH 7.4 aqueous medium under accelerated conditions provided by a temperature of 70 °C. It is clear that all samples were hydrolytically degradable since the weight loss after 60 days of exposure ranged between 31% and 48%. Degradation obviously proceeded at a slower rate when the temperature was decreased to 37 °C. In this case, weight loss values were lower than 5% (data not shown). The three copolyesters were different in terms of their degradation. In aliphatic/aromatic copolyesters, melting behaviour is mainly determined by the length of aromatic sequences in the polymer chains, which depends both on the composition and the structure. Besides the fixation of the polymer chains in the crystalline domains, the flexibility of the chain itself also influences the degradation behaviour to some extent. Hydrolytic and enzymatic degradation of poly(butylene succinate) and its copolymers have recently been reviewed, emphasizing the great influence of the low flexibility of polymer chains on their relatively slow hydrolysis rate [31]. Therefore, copolyesters with long aliphatic dicarboxylic acids exhibit a somewhat higher degradation rate than those with shorter ones [10]; however, this effect usually is masked by the much higher influence of the melting point. Degradation products of poly(butylene succinate-*co*-terephthalate) after enzymatic hydrolysis have been analysed and the water-soluble oligomers up to hexamer were detected after hydrolysis. The oligomeric fragments were slowly hydrolysed by secondary hydrolysis into 4-hydroxyl succinate and terephthalate [32]. Therefore, contrary to expectations about the length of the dicarboxylic content, the lowest weight loss for the sebacate derivative could be attributed to the higher insolubility of the degradation fragments.

Incorporation of HAp caused an increase in degradability compared to the parent copolyester, which could be explained by the increase in hydrophilicity. After 60 days of exposure, the weight loss increased by 4–7% with respect to the parent copolyester, where the higher percentage was related to the less degradable PBSeT-HAp sample. It seems that the effect caused by the hydrophilic HAp particles was more relevant when the polymer matrix was more hydrophobic.

**Figure 12:**
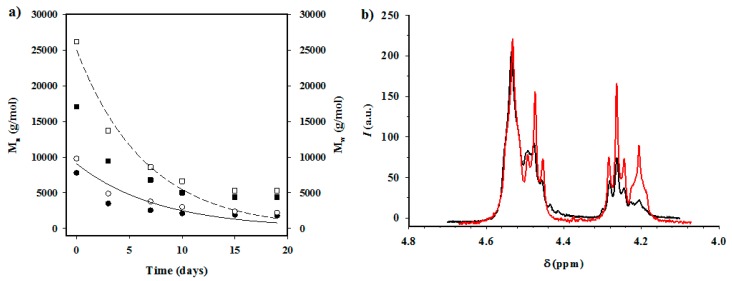
(**a**) Plot of the variation of the number (solid line) and weight (dashed line) average molecular weight of PBAdT and PBAdT-HAp samples during exposure time to the hydrolytic medium at 70 °C as simulated considering a first-order degradation mechanism. Experimental data (squares and circles for *M*_w_ and *M*_n_, respectively) taken during degradation are shown for both the copolyester (full symbols) and the nanocomposite (open symbols); (**b**) ^1^H NMR spectrum of a PBAdT samples before (red line) and after exposure (black line) for 60 days to the hydrolytic medium at 70 °C.

Average molecular weight during exposure to the 70 °C medium is shown in Figure 12a for PBAdT and PBAdT-HAp samples. In this case, the degradation process is more evident than that observed from weight loss measurements. In fact, molecular weight rapidly decreased, reaching a practically constant *M*_n_ value of 1200–750 g/mol after only 19 days of exposure. In this case, the differences between copolyesters and their nanocomposites were scarce (data not shown), due to the rapid change in the molecular weight.

A first-order degradation mechanism was assumed to quantify and compare the degradation rate of different samples. In fact, hydrolytic degradation of polyesters in the early stages has been usually simulated based on the exposure time dependence of molecular weight given by Equation (5) [33,34]:

*M* = *M*_0_ e^−*kt*^ (7)

where *M* is the molecular weight at time *t*, *M*_0_ is the initial molecular weight, and *k* indicates the kinetic constant.

Figure 12a shows a good fit between the experimental and simulated data for both number and weight average molecular weights when the exposure time was low, and a clear deviation at times longer than seven days. It is clear that molecular weight tended to asymptotic values, which should be related to the molecular size required to get soluble fragments. Obviously, the kinetic approximation was not correct for high exposure times.

Values of kinetic constants clearly showed an increase when HAp was incorporated. Specifically, the values determined for PBAdT increased from 0.039 days^−1^ to 0.048 days^−1^ when the number average molecular weights were considered, and from 0.045 days^−1^ to 0.055 days^−1^ when *M*_w_ data were used.

Analysis of the NMR spectra of degraded samples (Figure 12b) showed a great decrease in the peak associated with the ABA sequence, demonstrating that degradation mainly occurred through the aliphatic units, as might be presumed.

**Figure 13:**
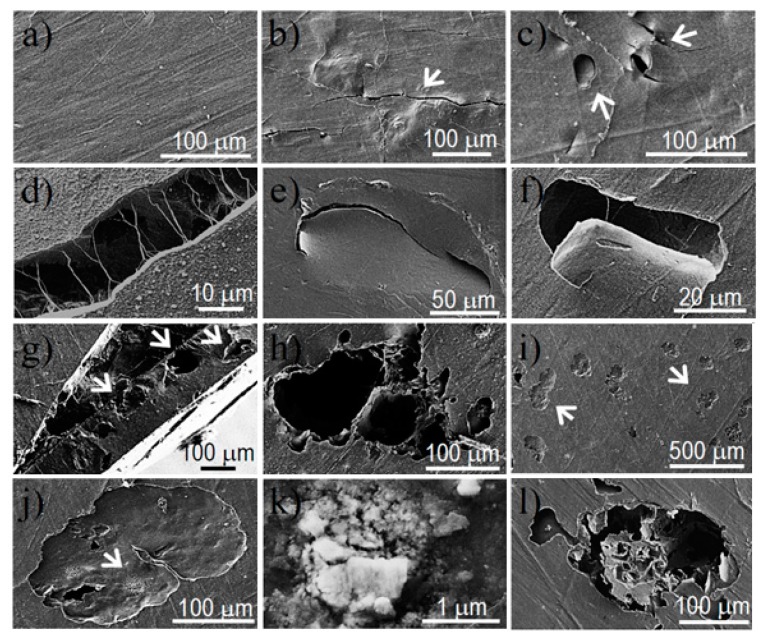
SEM micrographs showing the surface of PBAdT films at the beginning (**a**) and after exposure to a pH 7.4 aqueous medium at 70 °C for 60 days (**b**–**h**) and to a *Pseudomonas cepacia* enzymatic medium for 52 days (**i**–**l**). Arrows pointed out significant cracks and holes.

SEM micrographs revealed that hydrolytic degradation mainly took place in the inner parts of the exposed films due to a local concentration of acidic degradation products. Figure 13 shows the initial film surface of the PBAdT representative sample and details of films after exposure for 60 days to the hydrolytic medium. It is clear that the initial smooth surface (Figure 13a) evolves into the formation of cracks (Figure 13b) with interconnecting fibres (Figure 13d) and sinking of the surface (Figure 13c,e,f). This is caused by the development of deep inner holes (Figure 13g) that end up affecting the surface appearance (Figure 13h).

### 3.8. Enzymatic Degradation

**Figure 14:**
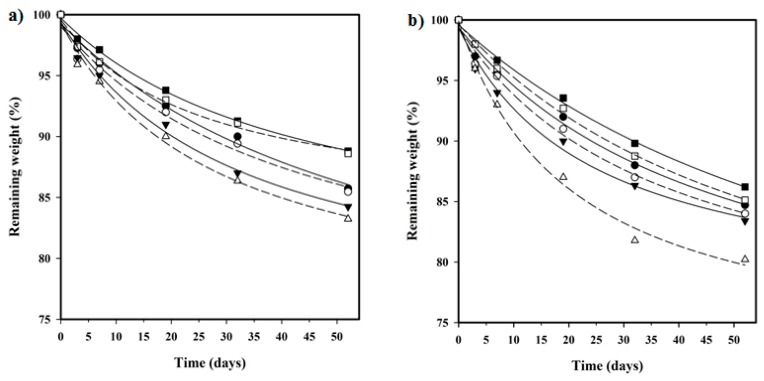
Plot of the variation in the remaining weight percentage during exposure time to enzymatic media of lipases from *porcine* (**a**) and *Pseudomonas cepacia* (**b**) for succinic (circles), adipic (triangles), and sebacic acid (squares) copolyesters with (open symbols) and without (full symbols) HAp.

Enzymatic degradation studies indicated that the three copolymers were susceptible to lipase attack (Figure 14). The degradation rate was slow but steadily progressed with exposure time. In general, the weight loss was between 16.5% and 15.5% after 55 days of exposure to *Pseudomonas cepacia*, the values being slightly lower for the porcine lipase medium (i.e., from 15.5% to 11%). In any case, trends were similar and specifically the highest and lowest weight losses were found for the adipic and the sebacic acid derivatives, respectively. Results are in agreement with those attained for hydrolytic degradation and probably are not a consequence of a specificity of selected enzymes towards the polymer substrate. Interestingly, the incorporation of HAp nanoparticles increased the enzymatic degradability of all samples, with the effect being more pronounced for the adipic derivative. Thus, in this case, weight loss increased from 16.5% to 20% after 55 days of exposure.

The progress of degradation was also examined using SEM and the micrographs corresponding to large exposure times (Figure 13i–l) showed different degrees of surface erosion. This could lead to concavities (Figure 13i,j) where in some cases, degradation products could be retained (Figure 13k), and deep holes could justify the observed weight losses (Figure 13l). These eroded surfaces contrast with the smooth appearance of the initial films (Figure 13a).

Contact angle measurements on the surface of exposed films revealed a significant increase in the hydrophilicity, consistent with the degradation process. For instance, 30°, 44°, and 64° angles (not shown) were determined for PBAdT-HAp, PBST-Hap, and PBSeT-HAp samples after exposure for 55 days, respectively. In this way, the surface of films was enriched with hydrophilic terminal groups generated by the degradation process. Furthermore, the reduction in the contact angle from the values previously determined for non-exposed samples (i.e., 78.3°–72°, as shown in Figure 8) suggests that adipic and sebacic acid derivatives were the most and less degraded samples, respectively.

## 4. Conclusions

Poly(butylene terephthalate-*co*-aliphatic dicarboxylate)s having succinate, adipate, or sebacate acid units as aliphatic moieties could be obtained with a random distribution from the corresponding aromatic and aliphatic prepolymers (i.e., PBT and PBA). Transesterification reactions took place at 250 °C in a sufficient extension to render a random microstructure, as detected by NMR sequence analyses.

Incorporation of HAp nanoparticles in the reaction medium gave rise to grafting reactions between the hydroxyl groups placed on the HAp surface and the carboxyl terminal groups of polymer chains. A considerable fraction of polymer became insoluble due to the crosslinking process, whereas the soluble fraction revealed a significant increase of molecular weight as a consequence of the grafting reactions.

HAp had a clear influence on the thermal stability and specifically caused a stabilization that could reflect the decrease in terminal carboxylic groups as a consequence of the grafting reactions.

Incorporation of nanoparticles increased the elastic modulus of the samples and, more interestingly, their biocompatibility. HAp also increased the hydrophilicity of the sample surface, which is a crucial factor that allowed for enhancing both enzymatic and hydrolytic degradability. The degradation rate slightly varied depending on the aliphatic dicarboxylic unit. Specifically, adipic and sebacic acid derivatives gave rise to the highest and lowest rates in all assayed media, respectively.

## Figures and Tables

**Table 1:** GPC molecular weight data and polymerization yields.

Copolymer	Yield (%)	COOH (meq/g)	*M*_n_ (g/mol)	*M*_w_ (g/mol)	PDI
PBST	89%	17	9540	20,200	2.12
PBAdT	85%	31	7800	17,100	2.19
PBSeT	90%	25	7000	17,100	2.44
PBST-HAp	91%	15	9500	23,100	2.43
PBAdT-HAp	88%	23	9800	26,200	2.67
PBSeT-HAp	90%	23	9000	22,000	2.44

**Table 2:** Chemical shifts and assignments of the ^1^H NMR spectra of synthesized copolyesters.

Sequence	Chemical Shift (ppm)		
	PBST	PBAdT	PBSeT
**–C_6_H_4_**	8.14	8.17	8.14
**T–OC*H_2_*CH_2_CH_2_CH_2_O–T**	4.52	4.57	4.64
**T–OC*H_2_*CH_2_CH_2_CH_2_O–A**	4.46	4.52	4.59
**A–OC*H_2_*CH_2_CH_2_CH_2_O-T**	4.28	4.31	4.37
**A–OC*H_2_*CH_2_CH_2_CH_2_O–A**	4.20	4.25	4.31
**COC*H_2_*–…**	2.78	2.52	2.42
**T–OCH_2_C*H_2_*CH_2_CH_2_O–T**	2.05	2.08	2.06
**T–OCH_2_C*H_2_*C*H*_2_CH_2_O–A**	1.91	1.92	1.92
**A–OCH_2_C*H_2_*CH_2_CH_2_O–A**	1.76	1.80	1.79
**COCH_2_C*H_2_*–…**	-	1.72	1.64, 1.32

**Table 3:** Composition and sequence-distribution analysis of synthesized copolyesters.

Coplymer	Composition molar fraction		Fraction of diads centered in the butylene units				Probability of finding units		Block lengths		Degree of randomness
	*f*_T_	*f*_A_	*f*_TT_	*f*_TA_	*f*_AT_	*f*_AA_	*P*_TA_	*P*_TA_	*L*_nTB_	*L*_nAB_	*r*
PBST	0.65	0.35	0.40	0.24	0.24	0.12	0.38	0.65	2.63	1.53	1.03
PBAdT	0.64	0.36	0.38	0.25	0.25	0.12	0.39	0.61	2.56	1.64	1.00
PBSeT	0.65	0.35	0.45	0.22	0.22	0.11	0.34	0.70	2.94	1.43	1.04

**Table 4:** Calorimetric data for the studied copolyesters and nanocomposites ^a^.

Sample	1st Heating run			Cooling run		2nd Heating run			
	*T*_m_ (°C)	Δ*H*_f_ (J/g)	*χ*^α^ (%)	*T*_c_ (°C)	Δ*H*_c_ (J/g)	*T*_g_ (°C)	*T*_m_ (°C)	Δ*H*_f_ (J/g)	*χ*^α^ (%)
PBST	153, 159	19.6	21	119	15.9	−7	155, 159	20.8	22
PBAdT	153, 163	21.0	23	131	18.4	−27	159	23.9	26
PBSeT	159, 163	23.7	26	128	19.1	−27	164	20.6	22
PBST-HAp	149, 159	19.1	20	119	17.4	−7	158	21.9	23
PBAdT-HAp	158, 164	19.6	22	126	19.5	−14	163	23.8	26
PBSeT-HAp	160, 166	22.7	26	129	22.3	−27	162	23.4	27

**^a^** Degree of crystallinity of the aromatic phase estimated from the melting enthalpy reported for a 100% crystalline PBT sample (∆*H*^100^) and the experimental composition determined by ^1^H NMR (i.e., *χ* = Δ*H*/(Δ*H*^100^ × *m_T_*), *m_T_* being the mass fraction of the BT repeat unit).

**Table 5:** TGA and DTGA data for thermal decomposition of the studied copolyesters and nanocomposites.

Sample	*T*_onset_ (°C)	*T*_peak_ (°C)	Char yield (%)
PBST	315	402	2.8
PBAdT	318	405	5.6
PBSeT	322	405	5.2
PBST-HAp	352	405	5.6
PBAdT-HAp	342	404	7.9
PBSeT-HAp	348	407	6.3

**Table 6:** DMTA data of the studied copolyesters and nanocomposites ^a^.

Sample	*T*_g_ (°C)	*E’*_−20 °C_ (MPa)	*E’*_20 °C_ (MPa)	*E’*_70_ (MPa)
PBSTS	10.5	1830	480	180
PBAdT	−2.5	1840	550	250
PBSeT	−6.5	2030	370	80
PBST-HAp	11.0	2210	590	220
PBAdT-HAp	2.3	2470	700	290
PBSeT-HAp	−3.5	2200	400	100

**^a^** Storage moduli are given for temperatures below (−20 °C) and higher than (20 °C) the first glass transition temperature and after the second glass transition temperature (70 °C).
